# Planar Microwave Sensing Technology for Soil Monitoring

**DOI:** 10.3390/s26082509

**Published:** 2026-04-18

**Authors:** Salman Alduwish, Yongxiang Li, James Scott, Akram Hourani, Nasir Mahmood

**Affiliations:** 1 Department of Electrical and Electronic Engineering, School of Engineering, STEM College, RMIT University, Melbourne, VIC 3000, Australia; james.scott@rmit.edu.au (J.S.); akram.hourani@rmit.edu.au (A.H.); 2 School of Science, STEM College, RMIT University, Melbourne, VIC 3000, Australia; nasir.mahmood@rmit.edu.au

**Keywords:** planar MW sensors, high resolution, soil properties, precision agriculture, sensitivity, microstrip patch antennas, split-ring resonators, complementary split-ring resonators, multi-parameter sensing

## Abstract

Planar microwave (MW) sensors offer high-resolution, non-invasive technology for monitoring critical soil properties, serving as a support for modern precision agriculture. While laboratory studies confirm their exceptional sensitivity, the widespread adoption of these sensors is severely impeded by critical translational challenges that constitute a defining “lab-to-field gap”. These barriers include high sensor-to-sensor variability, debilitating thermal cross-sensitivity, soil heterogeneity necessitating unique site-specific calibration, and the enduring tension between high-performance and cost-effective scaling. This review systematically synthesizes the current state of planar permittivity MW technology, moving beyond technical mechanisms to critically assess these operational limitations. We detail advanced architectural strategies designed to bridge this gap, focusing particularly on the transition toward more robust solutions. The key strategies analyzed include the adoption of differential sensor designs using microstrip patch antennas to mitigate common-mode environmental errors, the integration of ultra-compact metamaterial structures such as split-ring resonators (SRRs) and complementary split-ring resonators (CSRRs) for enhanced field robustness and deep soil sensing, and the necessity of multi-parameter sensing capabilities (moisture, pH, and salinity). By establishing a comprehensive roadmap that prioritizes field stability, cost efficiency, and seamless IoT integration, this review demonstrates that planar MW sensors are poised to become reliable and scalable tools. Addressing these critical translational hurdles will ensure optimal resource management, significantly enhance crop productivity, and enable sustainable practices within smart farming ecosystems.

## 1. Introduction

Planar microwave (MW) soil sensors have emerged as a valuable technology for non-invasive and precise soil characterization, providing a means to assess critical properties such as moisture content, pH value, and salinity [1,2,3]. While large-scale techniques like satellite microwave remote sensing provide broad coverage, these systems typically suffer from limitations such as coarse spatial resolution and sensing depth (often limited to the top 5 cm of soil), and significant systematic differences from ground-truth measurements. In contrast, planar permittivity sensors are designed as in situ technologies, offering high-resolution, localized measurements essential for effective precision agriculture.

These sensors are vital for optimizing agricultural practices, ensuring sustainable resource management, and enhancing environmental monitoring [2,3,4]. However, the adoption of MW soil sensors in precision agriculture faces challenges related to sensor sensitivity, selectivity, and integration into existing agricultural systems.

Among the various MW sensor types, planar permittivity sensors have gained significant attention because of their compact design, low cost, and ability to capture high-quality data [4,5,6,7,8,9]. These sensors offer advantages over traditional soil sampling methods in terms of real-time monitoring capabilities and reduced labor requirements. Planar sensors are favored for their versatility and ease of integration into the existing systems. Their flat and compact structure allows for flexible deployment in various soil environments, making them ideal for in situ measurements. Additionally, planar sensors can be easily manufactured using standard printed circuit board techniques, thereby reducing production costs and enabling mass production for widespread use in precision agriculture [10,11,12,13,14,15,16,17,18,19].

Recent developments in MW soil sensing have focused on improving the sensitivity and accuracy of the planar sensors. Techniques such as microstrip technology and metamaterials have significantly enhanced the sensitivity and selectivity of these sensors, thereby opening new possibilities for real-time soil monitoring. The unique electromagnetic properties of microwaves, particularly their ability to penetrate soil and interact with its components, drive these advancements, allowing for the precise measurement of soil dielectric properties that directly correlate with factors such as soil moisture levels [20,21,22,23,24,25,26,27,28,29,30].

While the existing literature [30,31,32,33,34,35,36,37,38,39,40,41,42,43,44,45,46,47,48,49,50,51,52,53,54,55,56,57,58,59,60] has predominantly focused on the technical development of planar permittivity MW sensors, less attention has been given to their practical implementation and the challenges faced in real-world agricultural settings. This review aims to bridge this gap by critically assessing the development of planar permittivity MW sensors, identifying the challenges in their application for soil monitoring, and proposing future research directions to enhance their practical utility in precision agriculture.

Specifically, this literature review explores the current state of planar permittivity MW sensing technology for soil applications by examining the principles behind its operation, different types of planar sensors, and their specific applications in soil property monitoring. It also discusses recent advancements in planar sensor design and fabrication, highlighting the improvements in sensitivity, accuracy, and reliability. Furthermore, this review addresses the challenges and limitations of planar permittivity MW soil sensors and outlines future research directions aimed at overcoming these obstacles and enhancing their capabilities.

### Review Methodology

The literature search was conducted across multiple electronic databases and platforms, including IEEE Xplore, Scopus, Web of Science, and Google Scholar. The search utilized a combination of keywords and Boolean operators tailored to capture relevant studies on planar microwave sensors for soil monitoring. Key search terms included “planar microwave sensors,” “soil moisture monitoring,” “split-ring resonators,” “complementary split-ring resonators,” and “microstrip patch antennas.” The search was limited to publications available up to 2025, ensuring coverage of the most recent advances in the field.

## 2. MW Sensing

### 2.1. MW Sensing Mechanism

The principle of MW sensing is predicated on the interaction of electromagnetic waves with the tested sample through a sensing structure. The fundamental material property characterized is permittivity (ε), which is a physical quantity that describes the ability of a material to store and dissipate energy in an electromagnetic field. The absolute permittivity (ε) of a material is defined in relation to the permittivity of free space ($\varepsilon_{0}$) and the complex relative permittivity ($\varepsilon_{r}$) [31]:

(1) $$\varepsilon ={\varepsilon_{r}\times \varepsilon }_{0}$$

The complex relative permittivity, $\varepsilon_{r}$, is then expressed as follows:

(2) $$\varepsilon_{r}={\varepsilon_{r}}^{\prime }-j\varepsilon_{r}\prime \prime$$

where ${\varepsilon_{r}}^{\prime }$ is the real part of the complex relative permittivity, also known as the dielectric constant, and ${\varepsilon_{r}}^{\prime \prime }$ is the imaginary part of the complex relative permittivity, which is proportional to the energy loss. The imaginary permittivity is directly related to the RF conductivity σ of the material, representing how electromagnetic energy is dissipated as heat due to conduction losses. This relationship is expressed by the following equation:

(3) $${\varepsilon_{r}}^{\prime \prime }=\frac{\sigma }{\omega \varepsilon_{0}}$$

where ω=2πf is the angular frequency of the electromagnetic wave. This equation shows that the imaginary part of the permittivity can be interpreted as a measure of the material’s conductive losses at a given frequency. Expressing losses in terms of σ is often preferred in RF and microwave engineering because conductivity intuitively describes how free charges contribute to energy dissipation. Additionally, the dielectric loss tangent of a material can be calculated as follows [31]:

(4) $$tan\;\delta =\frac{{\varepsilon_{r}}^{\prime }}{{\varepsilon_{r}}^{\prime \prime }\;}$$

Different materials exhibit a wide range of permittivities, which are intrinsic properties that describe how a material interacts with electric fields. In mixtures, the overall permittivity is influenced not only by the individual permittivities of each component but also by their spatial arrangement and structural configuration within the mixture. Understanding the relationship between a material’s permittivity and its composition allows for the characterization of its physical and chemical properties. This correlation is critical for detecting subtle changes in material parameters, which can be indicative of variations in composition, phase transitions, or other material alterations.

Microwave (MW) sensing leverages this principle by using the interaction between electromagnetic (EM) waves at microwave frequencies—typically ranging from 300 MHz to 300 GHz—and the sample under test. The sensing structure facilitates this interaction, enabling the measurement of how the EM waves are affected by the material’s permittivity. Compared to lower frequency ranges such as radio frequencies (RFs), MW frequencies provide higher resolution and sensitivity due to their shorter wavelengths, allowing for more precise detection of small changes in material properties. Additionally, microwaves can penetrate many materials effectively, enabling non-destructive testing, while still being manageable in terms of instrumentation complexity.

**Figure 1:**
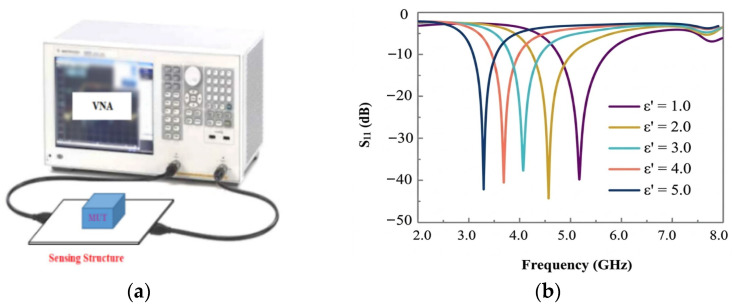
MW sensing system: (**a**) measurement setup with sensor loaded by material under test (MUT); (**b**) sensor response.

A vector network analyzer (VNA) serves as the primary instrument in this process, generating a low-power stimulus signal (typically less than 1 mW) and capturing the sample’s response in terms of scattering parameters (S-parameters). These S-parameters, referenced to a standard impedance of 50 Ω, quantify reflection (S_11_) and transmission (S_21_) characteristics of the sample. Depending on its configuration, a VNA can operate with one port to measure reflection only or with two ports to assess both reflection and transmission, providing a comprehensive electromagnetic characterization of the material’s properties (Figure 1).

### 2.2. MW Sensing Classification

MW sensors have been extensively employed in the characterization of the dielectric properties of various materials such as soil. These sensors operate based on the interaction between the MW signal and the dielectric properties of the material [33]. Two principal working principles are frequency variation (detects shifts in a single resonant mode), and frequency splitting (exploits induced asymmetry to produce and measure the separation between two resonant modes).

#### 2.2.1. Frequency-Variation MW Sensors

MW sensing, based on variations in the resonant frequency, has been extensively studied in the literature for measuring the dielectric properties of materials by observing changes in the sensor’s resonant frequency.

**Figure 2:**
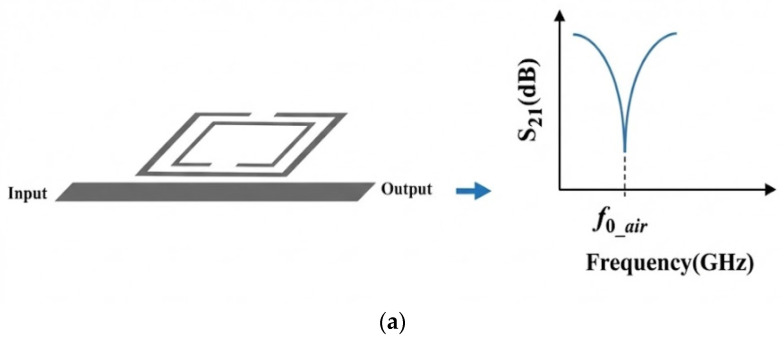

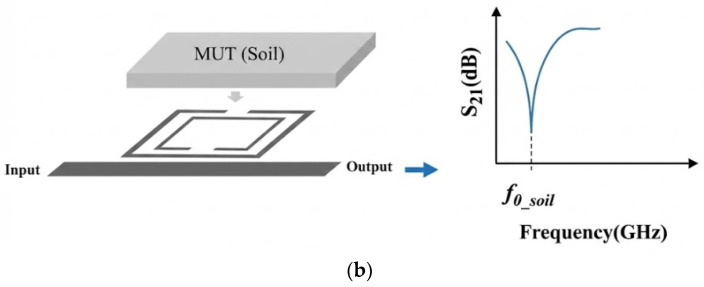
Frequency-variation principle for MW soil sensor: (**a**) unloaded and (**b**) loaded (MUT).

Figure 2 demonstrates how the presence of soil in proximity to a planar microwave (MW) resonator significantly influences its resonant frequency. In the absence of soil, the resonator maintains a characteristic resonance at its original frequency, as evidenced by the sharp peak displayed in the graph. This baseline frequency is determined by the intrinsic physical and electrical properties of the resonator itself. However, when soil with distinct dielectric properties is introduced onto or near the resonator surface, the electromagnetic environment surrounding the device changes. The soil’s dielectric constant effectively modifies the local permittivity experienced by the resonator, thereby altering its electromagnetic field distribution and energy storage characteristics [33].

This modification manifests as a measurable downward shift in the resonant frequency, as shown in the right-hand graph of Figure 2. The decrease in frequency occurs because the soil’s dielectric constant increases the effective permittivity, which in turn increases the resonator’s capacitance component, lowering the natural frequency of oscillation. This shift is a critical parameter for sensing applications, as it enables the resonator to detect and quantify soil properties such as moisture content, composition, or contamination levels. The sensitivity of the resonant frequency to the soil’s dielectric properties highlights the resonator’s potential utility in environmental monitoring, agriculture, and geotechnical engineering, where non-invasive, real-time soil characterization is essential.

The mechanism of frequency variation in MW sensors for soil moisture detection is based on shifts in the resonance frequency caused by changes in soil dielectric properties. These sensors are sensitive to the dielectric constant and dielectric loss of materials, as described by the following equations:

(5) $$f_{0\_air}=\frac{1}{2\pi \sqrt{LC}}$$

When soil is introduced into the resonator, it increases the capacitance and alters the resonant frequency.

(6) $$f_{0\_soil}=\frac{1}{2\pi \sqrt{LC\prime }}$$

(7) $$C^{\prime }=C+C_{soil}$$

where L is the inductance of the MW sensor, *C* is its capacitance without the soil present, and $C_{soil}$ represents the additional capacitance due to the soil.

The resonant frequency shift ($f_{0\_air}$ > $f_{0\_soil}$) correlates with the soil dielectric properties, particularly the permittivity. As soil moisture increases, its permittivity increases, causing a more pronounced frequency shift. This relationship forms the basis for soil moisture content measurements using MW sensors. The sensitivity of the sensor can be inferred from the magnitude of the frequency shift, with larger shifts for a given soil moisture level indicating greater sensitivity in the sensor.

The full complex relative permittivity can be analytically calculated from the resonant response by measuring two independent variables: the shift in the resonant frequency ($∆f_{0}$) and the change in the quality factor (ΔQ), (or the magnitude of the resonance ($∆\left|S_{21}\right|$)). The real part ($\varepsilon_{r}\prime$) of the complex relative permittivity is predominantly related to the frequency shift ($∆f_{0}$), while the imaginary part (ε″) of the complex relative permittivity is related to energy absorption, causing changes in ΔQ or the magnitude of the measured S-parameter. This complex relationship can be formally solved using a linear matrix model:

(8) $$\left(\begin{array}{c} ∆f_{0}\\ ∆\left|S_{21}\right| \end{array}\right)=\left(\begin{array}{cc} m_{11} & m_{12}\\ m_{21} & m_{22} \end{array}\right)\left(\begin{array}{c} ∆\varepsilon_{r}\prime \\ ∆\varepsilon_{r}\prime \prime \end{array}\right)$$

where $∆f_{0}$ and $∆\left|S_{21}\right|$ are the measured changes in resonant frequency and peak attenuation, respectively, caused by the change in the complex relative permittivity ($∆\varepsilon_{r}\prime$ and $∆\varepsilon_{r}\prime \prime$) of the material. The coefficients $m_{ij}$ are sensor-dependent parameters determined by initial calibration using reference samples with known complex permittivity values. This methodology allows for the comprehensive extraction of the complex relative permittivity.

Alternatively, for empirical extraction of the complex relative permittivity ($\varepsilon_{r}$), especially when the loss factor is considered negligible, the relationship between the soil measured resonant frequency ($f_{0}$) and the relative permittivity can be approximated using a Curve Fitting Model, either linear (Equation (9)) or polynomial (Equation (10)):

(9) $$\varepsilon_{r}=af_{0}+b$$

(10) $$\varepsilon_{r}=af_{0}^{2}+bf_{0}+c$$

where the coefficients a, b, c can be found by using standard Materials Under Test (MUTs) to generate resonant frequency data and using the known relative permittivities of those MUTs.

However, the relationship between frequency shift, soil permittivity, and moisture content is complex and often nonlinear. Researchers have typically developed empirical models or calibration curves to relate observed frequency shifts to soil moisture content for specific soil types, considering factors such as soil texture, organic matter content, and temperature [25].

#### 2.2.2. Frequency-Splitting MW Sensors

A frequency-splitting microwave (MW) sensor operates by exploiting the phenomenon of resonant frequency splitting, which occurs when a material with distinct dielectric properties interacts with the sensor’s resonant elements. This interaction causes the original resonance frequency to split into two closely spaced frequencies, the characteristics of which are directly influenced by the dielectric constant and loss tangent of the tested material. By precisely measuring these frequency shifts, the sensor can accurately characterize the material’s dielectric properties, making it highly effective for applications requiring sensitive and non-destructive evaluation of materials [33].

The sensor’s planar design typically incorporates resonant structures such as split-ring resonators (SRRs) and complementary split-ring resonators (CSRRs), which are engineered to produce strong electromagnetic fields localized near their surfaces. These resonators enhance the sensor’s sensitivity to changes in the dielectric environment by concentrating the electric field in regions where the test material is placed. The combination of SRRs and CSRRs not only improves the sensor’s ability to detect subtle variations in permittivity but also allows for compact and integrable sensor architectures suitable for diverse material testing scenarios, including quality control, biomedical sensing, and environmental monitoring.

**Figure 3:**
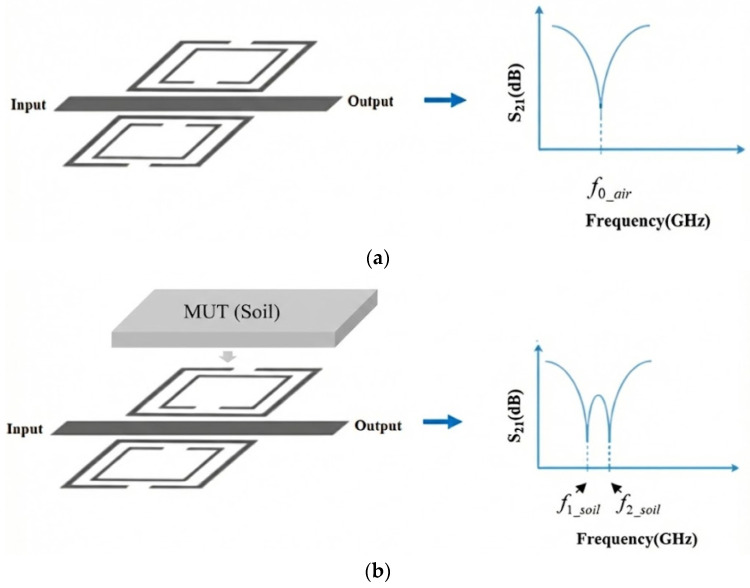
Frequency-splitting principle for MW soil sensor: (**a**) unloaded and (**b**) loaded (MUT).

Figure 3 illustrates the effect of the soil interaction on the frequency-splitting phenomenon in a planar split-ring resonator.

In the absence of a sample, the MW sensor [33] exhibited a single resonant frequency, which was determined by its geometric configuration and material composition. Under these conditions, the electromagnetic field distribution of the sensor remained symmetric, and the system supported a single transmission zero, representing its fundamental resonant mode.

When one of the sensing resonators is loaded with a dielectric sample, such as soil, the localized permittivity increases, perturbing the electromagnetic environment of the resonator. This asymmetry breaks field uniformity and results in the emergence of two distinct transmission zeros (resonances), each corresponding to a separate resonant mode. These modes are characterized by the following equations:

(11) $$f_{1\_soil}=\frac{1}{2\pi \sqrt{LC_{1}}}$$

(12) $$f_{2\_soil}=\frac{1}{2\pi \sqrt{LC_{2}}}$$

where $C_{1}$ and $C_{2}$ represent the effective capacitances of the two resonant sections, one perturbed by the sample and the other remaining in its reference state. The resulting difference between the split frequencies is defined as the frequency split (Δ*f*).

(13) $${∆f=f}_{2\_soil}-f_{1\_soil}$$

To extract the full complex relative permittivity using the frequency-splitting method, two independent output variables are typically measured: the frequency split (∆f) and the difference in magnitude of the two notches ($∆\left|S_{21}\right|$). The real part of the relative permittivity (ε′) is often correlated primarily with the frequency split, while the imaginary part (ε″) is related to the difference in the attenuation (magnitude) of the two notches. An analytical matrix model, similar to that used for frequency variation sensors, can be employed, where the input vector is the change in the complex relative permittivity (∆ε′) and ∆ε″)) and the output vector consists of ∆f and ${∆\left|S_{21}\right|}_{notches}$:

(14) $$\left(\begin{array}{c} ∆f\\ {∆\left|S_{21}\right|}_{notches} \end{array}\right)=\left(\begin{array}{cc} k_{11} & k_{12}\\ k_{21} & k_{22} \end{array}\right)\left(\begin{array}{c} ∆\varepsilon \prime \\ ∆\varepsilon \prime \prime \end{array}\right)$$

where $k_{ij}$ represents the sensor-specific coefficients. This approach allows for simultaneous determination of both the dielectric constant and the loss tangent of the material under test.

As an alternative empirical approach, the relationship between the measured frequency split (∆f) and the real part of the relative permittivity can be derived through a Curve Fitting Model:

(15) $$\varepsilon_{r}=a∆f+b$$

(16) $$\varepsilon_{r}=a{\left(∆f\right)}^{2}+b∆f+c$$

This frequency-splitting phenomenon serves as a key indicator of the dielectric loading asymmetry and is highly sensitive to the permittivity contrast introduced by the sample. The magnitude of ∆f provides valuable insights into the material properties of the test sample, most notably its moisture-dependent permittivity in the case of soils.

Accurate quantification and tracking of this split enables enhanced resolution in soil moisture estimation, leveraging symmetric structures for improved baseline stability and detection sensitivity.

### 2.3. MW Sensing Performance

Accuracy and sensitivity are paramount parameters in the design of MW sensors, fundamentally determining the performance and reliability of these devices. Accuracy is quantified primarily by the Quality factor (Q), which measures energy loss and signal selectivity, while sensitivity is determined by the magnitude of the frequency shift (∆f) in response to material changes. The design optimization of planar MW sensors is perpetually constrained by the intrinsic trade-off between maximizing sensitivity and ensuring high measurement accuracy, quantified by the Quality factor.

#### 2.3.1. Accuracy Analysis

**Figure 4:**
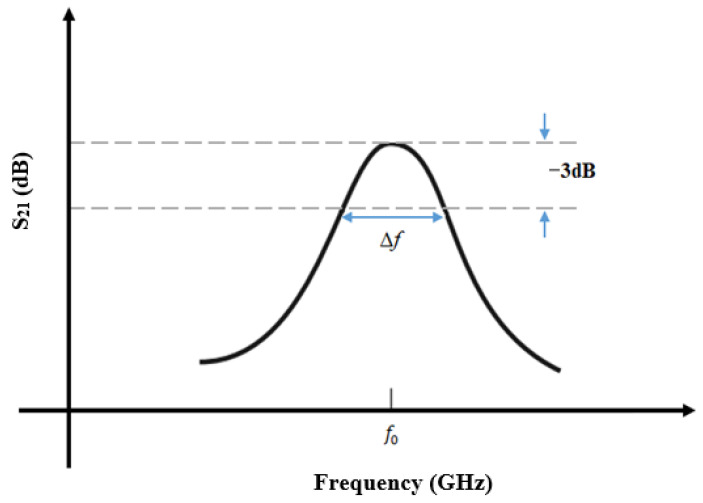
Measurement of quality factor from $S_{21}$ parameter.

Accuracy is an essential parameter for estimating the quality of MW sensors, where a higher Q-factor indicates lower energy losses and higher selectivity, which directly affect the accuracy of the sensor. The unloaded *Q*-factor can be calculated as follows [40]:

(17) $$Q=\frac{f_{0}}{∆f}$$

where ∆f is the bandwidth of the response at the −3 dB points, as shown in Figure 4. The *Q*-factor increases as the losses in the resonator decrease. Thus, a higher Q-factor leads to a narrower peak (or dip) versus frequency in the measured response. This then means that the resonant frequency, $f_{0}$ can be measured more precisely.

Figure 4 shows the measurement of the quality factor obtained from the $S_{21}$ parameter [51,52,53,54].

The sensor’s structural parameters—including conductor width, gap spacing within the split-ring resonator, substrate thickness, and resonator length—critically influence the circuit’s lumped inductance and capacitance, which in turn determine the resonant frequency and quality factor (Q-factor). The inductance is predominantly controlled by the resonator length, while decreasing the resonator gap increases capacitance, resulting in a lower resonant frequency and enhanced sensitivity (this term will be defined in next subsection). Furthermore, reducing the spacing between the resonators and the through microstrip line improves electromagnetic coupling, which further elevates the Q-factor and overall sensor sensitivity. Systematic optimization of inductor geometry and resonator-to-line spacing is therefore essential to precisely tune inductance, capacitance, coupling, and maximize sensor performance.

Accurate parameter tuning through geometric modifications enables maximizing the sensor’s responsiveness to dielectric perturbations while maintaining high-Q resonance. However, there are inherent trade-offs and practical limits to these adjustments. The inductance cannot be altered indefinitely, as it is primarily constrained by the physical length and geometry of the resonator. Similarly, the gap size in the resonator has a lower bound due to fabrication limits, restricting the maximum achievable capacitance. These constraints affect the resonator’s loss characteristics: excessively small gaps or extreme inductance values can increase conductor and dielectric losses, thereby degrading the Q-factor. Q-factor extraction using the transmission parameter illustrates that the sharpness and depth of the resonance curve directly reflect these losses and overall sensor quality. Therefore, optimizing sensor performance requires balancing inductance and capacitance tuning with practical fabrication limits and loss mechanisms to preserve a high Q-factor and sensitivity.

#### 2.3.2. Sensitivity Analysis

**Figure 5:**
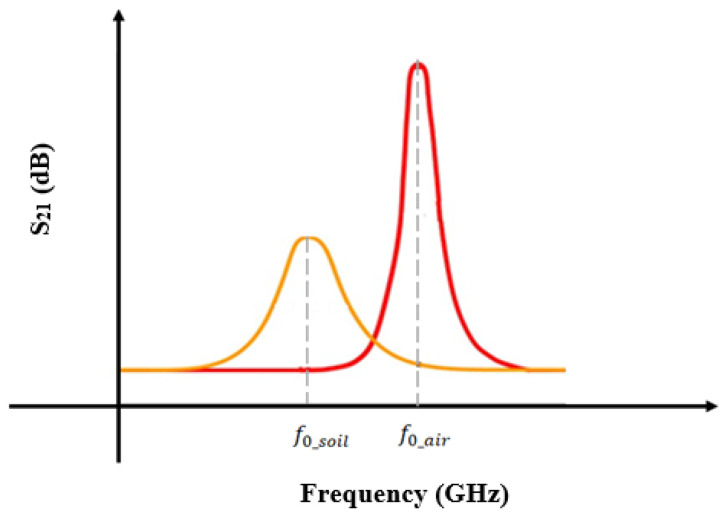
Change in MW properties when sensor is loaded with material.

The sensitivity of the microwave (MW) sensor is fundamentally characterized by observing the shift in resonance frequencies when transitioning from an air-filled state to one loaded with a material under test (MUT). The air sensor serves as a baseline or reference due to the well-established electromagnetic properties of air, specifically its relative permittivity and loss tangent, which are close to unity and negligible, respectively. When the MUT is introduced into the sensor’s region of strongest electric field intensity, it perturbs the local electromagnetic environment, causing a measurable shift in the resonance frequency. This shift is directly related to the dielectric properties of the MUT, particularly its relative permittivity, which influences how the electric field interacts with the material. These changes in microwave properties when the sensor is loaded with material are visually summarized in Figure 5.

This relationship between the resonance frequency ($∆f_{r})$ change and the MUT’s relative permittivity is linear, enabling precise quantification of the material’s dielectric constant by comparing the loaded sensor frequency to the air reference frequency. Since air’s relative permittivity is approximately 1, the difference in permittivity ($∆\varepsilon_{r}$) can be effectively represented as the difference between the real part of the MUT’s relative permittivity and that of air. This linear dependence allows for straightforward calibration and sensitivity analysis, making the sensor a reliable tool for detecting subtle variations in material properties, which is critical in applications requiring accurate dielectric characterization.

Therefore, the sensitivity S of the sensor can be determined as follows [55]:

(18) $$S=\frac{∆f_{r}}{∆\varepsilon_{r}}=\frac{∆f_{r}}{\varepsilon_{r}-1}$$

(19) $$∆f_{r}=unloaded\;\left(f_{0}\right)-loaded\left(f_{0}\right)$$

(20) $$∆f_{r}=f_{0\_air}-f_{0\_soil}$$

The real part of the relative permittivity for the loaded sample is denoted as $\varepsilon_{r}$, representing the resonance frequency of the air sensor $f_{0\_air}$ and the resonance frequency when the sensor is loaded with the sample (soil) $f_{0\_soil}$. To ensure a consistent comparison, sensitivity values must be normalized by the resonance frequency of the air sensor. This normalization is essential because sensors operating at higher frequencies tend to show larger absolute shifts in the notch frequency relative to changes in the dielectric constant. The expression for normalized sensitivity is provided as follows [55]:

(21) $$S=\frac{f_{0\_air}-f_{0\_soil}}{f_{0\_air}\;(\varepsilon_{r}-1)}\times 100\%$$

The structural geometry of the sensor, including resonator length, gap spacing in the split-ring resonator, conductor width, and substrate height, directly governs its effective inductance and capacitance. These parameters determine the baseline resonance frequency and influence the strength and distribution of the electric field, which dictate the level of interaction with the sample. Narrowing the gap in the split-ring resonator can intensify the local field strength, enhancing the sensitivity of the sensor to dielectric variations. Similarly, increasing the resonator length or area can provide a broader interaction surface, improving the response to bulk permittivity changes.

### 2.4. MW Sensing Configuration

#### 2.4.1. Antenna-Based MW Sensors

Patch antenna-based sensors have emerged as promising tools for monitoring soil moisture in smart agriculture applications. These sensors offer advantages, such as wireless operation, high sensitivity, and potential for miniaturization [34,35]). The principle behind these sensors is that changes in soil moisture affect the dielectric properties of the surrounding medium, which in turn influences the antenna’s resonant frequency and other parameters [37]). Different approaches have been explored to enhance the performance of patch-antenna sensors for soil moisture monitoring. For instance, some designs incorporate T-shaped slots to improve the sensitivity and distinguish between frost, ice, and water [38]). Others utilize high-dielectric-constant substrates to achieve miniaturization and a higher sensing sensitivity [39]. The integration of complementary spiral resonators has also been proposed to achieve ultra-compact designs operating at lower frequencies, which are less sensitive to variations in the volume of soil under test [59].

**Figure 6:**
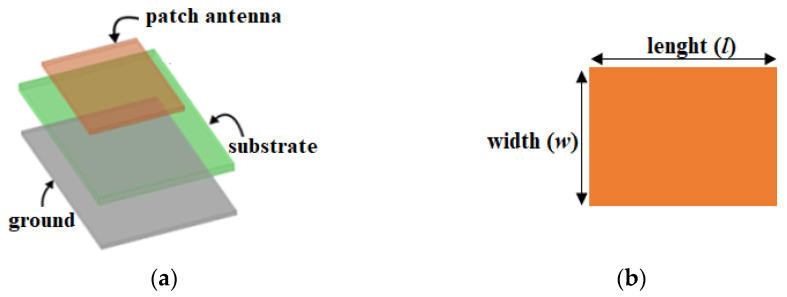
Microstrip patch antenna: (**a**) perspective view and (**b**) top view.

The resonant frequency is a fundamental parameter of an antenna that facilitates sensing functionality and is influenced by factors such as the physical size, shape, structure, and material/substrate of the sensor. As illustrated in Figure 6, the length (l) and width (w) of the antenna-based sensor are the primary parameters that directly affect the resonant frequency.

Equations (21) and (22) show that, as the length, width, or dielectric constant of the substrate increases, the center frequency ($f_{0}$) shifts towards a lower frequency and vice versa [40].

(22) $$w=\frac{c}{2f_{0}}\sqrt{\frac{2}{\varepsilon_{r}+1}}$$

(23) $$l=\frac{c}{2f_{0}\sqrt{\varepsilon_{reff}}}-0.412\left(\frac{\left(\varepsilon_{reff}+0.3\right)\left(\frac{w}{h}+0.264\right)}{\left(\varepsilon_{reff}-0.258\right)\left(\frac{w}{h}+0.813\right)}\right)$$

(24) $$\varepsilon_{reff}=\frac{\varepsilon_{r}+1}{2}+\frac{\varepsilon_{r}-1}{2}{\left(1+12\frac{h}{w}\right)}^{-0.5}$$

where c is the speed of light in vacuum (~3 × 10^8^ m/s).

Using this phenomenon, when a target material alters the antenna-based sensor’s resonant frequency, the interrogator module determines the material’s relative permittivity based on the frequency shift.

These sensors are highly sensitive to changes in material properties and are commonly used for applications where precise dielectric measurements are required [34,35,36,37,38,39].

**Figure 7:**
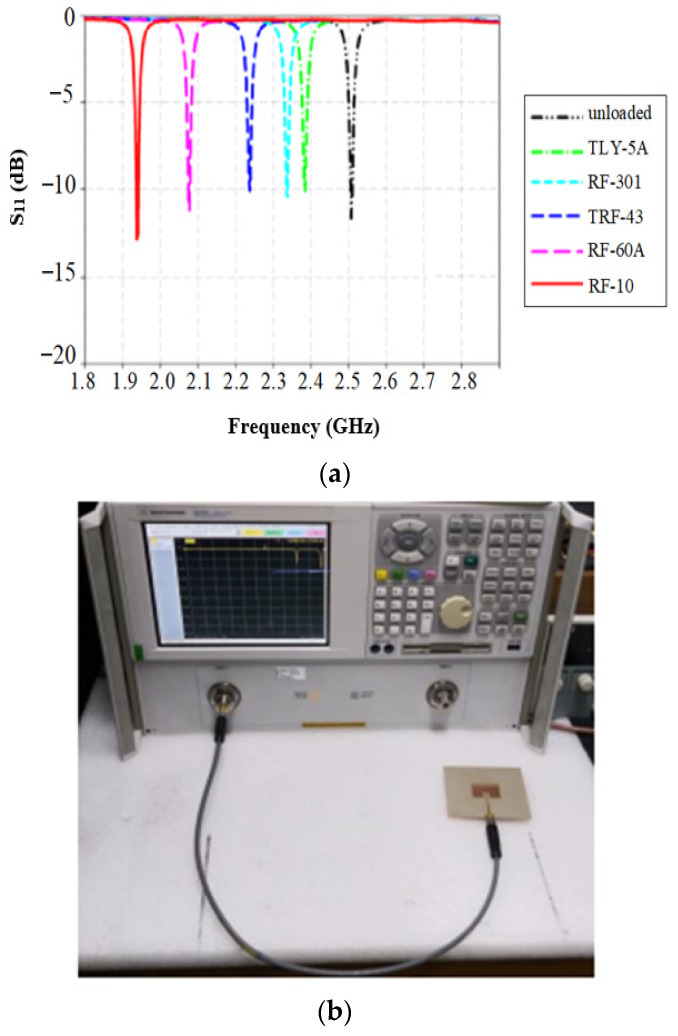
(**a**) MW sensing response based on frequency shift and (**b**) fabricated prototype and experimental setup [41].

Figure 7 illustrates how the resonant frequency shifts in response to variations in the permittivity of the material under test, demonstrating a clear example of frequency-based sensing using a patch antenna [41].

#### 2.4.2. Resonator-Based MW Sensors

Resonator-based sensors operate based on the principle of resonance, where they detect changes in a physical quantity by measuring the shifts in the resonance frequency or resonance characteristics of a sensing system. These sensors exploit the interaction between the resonator’s structure and its surrounding environment, which induces measurable changes in the resonant frequency. Recent advancements have aimed at enhancing the sensitivity, selectivity, and integration capabilities of these sensors across diverse applications. Microstrip resonator-based sensors use microstrip technology, characterized by conductive strips on a dielectric substrate, to sense changes in physical quantities by extracting complex permittivity. Among the commonly studied microstrip resonators are standard microstrip resonators, split-ring resonators (SRRs), and complementary split-ring resonators (CSRRs), as frequently documented in the literature [42,43,44,45,46,47,48].

**Figure 8:**
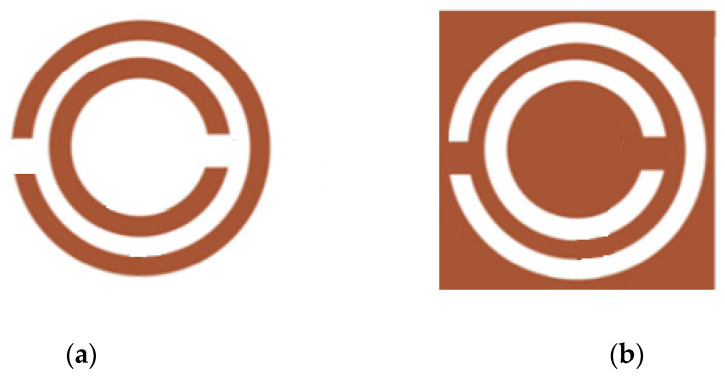
Microstrip resonator: (**a**) SRR and (**b**) CSRR. Source: Authors’ own work.

The use of these resonators with changes in the dielectric constant and frequency alteration has been investigated in various applications such as soil monitoring, as illustrated in Figure 8.

The operating principle of resonator-based sensors relies on shifts in resonant frequency caused by changes in effective permittivity when a sample interacts with the resonator. While this principle has been detailed in Section 2.2, here the focus shifts to comparing Split-Ring Resonators (SSRs) and Complementary Split-Ring Resonators (CSRRs) in terms of their characteristics and performance.

SSRs and CSRRs (Figure 8) differ primarily in their structure and coupling mechanisms. SSRs are metallic rings with gaps that behave as inductive–capacitive elements, while CSRRs have their negative images etched in the ground plane, creating complementary resonant behavior. CSRRs typically exhibit stronger electric field confinement and higher sensitivity to dielectric changes due to their planar structure and enhanced coupling with the microstrip line.

**Figure 9:**
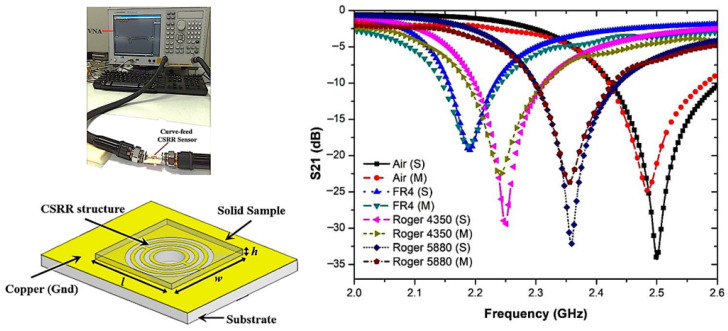
MW resonator-based sensor and its fabricated prototype and experimental setup. Reproduced from [50].

Figure 9 illustrates how the resonant frequency shifts in response to variations in the permittivity of the material under test, demonstrating frequency-based sensing using a hexagonal split-ring resonator [50]. Since the sample covers both resonators simultaneously, the sensor operates with a single resonance per MUT, indicating that it is not a differential sensor.

### 2.5. Recent Development in Planar MW Sensing for Soil Moisture Monitoring

Different configurations, such as microstrip patch antennas, microstrip ring resonators, split-ring resonators, and complementary split-ring resonators, have been widely investigated for applications in permittivity-based sensing, including soil measurement, owing to their high sensitivity, compact size, and ease of fabrication.

In smart agriculture, these sensors offer critical insights into soil properties such as moisture content, allowing farmers to optimize irrigation and fertilization strategies. The evolution of MW sensing technology has led to improved sensor performance and functionality. Researchers have developed sensors with enhanced sensitivity and accuracy, capable of detecting changes in soil moisture content on the order of 0.1% or less, enabling precise monitoring of subtle environmental fluctuations [21,22,23,24,25,26,27].

Moisture content/humidity monitoring plays a crucial role in optimizing processes and ensuring product quality in diverse industries. In agriculture, precise soil moisture measurements enable farmers to implement efficient irrigation strategies, conserve water resources, and enhance crop yields. Similarly, in food processing, accurate determination of moisture content is vital for maintaining product consistency, extending shelf life, and meeting regulatory standards. Traditional moisture measurement techniques often involve time-consuming and destructive methods, which are impractical for real-time monitoring in industrial settings [61,62].

#### 2.5.1. Soil Moisture Antenna-Based Sensors

This section focuses on recent developments in soil moisture sensing techniques, particularly those that use microstrip patch antenna designs. The sensors discussed operate on the principle of measuring changes in the dielectric properties of soil or plant material due to variations in water content. These changes were detected through shifts in resonant frequency.

The antenna-based sensors presented in this section include modified slotted microstrip patch antennas for leaf moisture measurement and circular microstrip patch antennas integrated with complementary split-ring resonators (CSRRs) for soil water content characterization. These designs demonstrated improved sensitivity compared to conventional microstrip patch antennas, compact form factors, and the ability to measure a wide range of moisture levels in different soil types and plant materials

**Figure 10:**
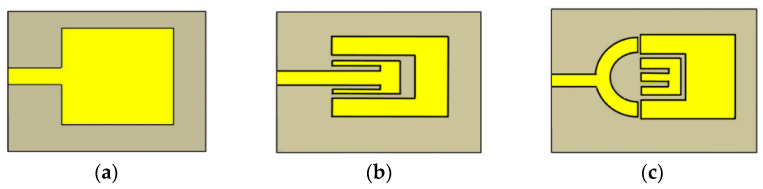
Microstrip patch antenna structures: (**a**) standard rectangular patch antenna, (**b**) slotted patch antenna, and (**c**) enhanced coupling modified slotted patch antenna [21].

Among recent innovations in this field, Khan et al. [21] introduced a modified slotted microstrip patch antenna designed for enhanced leaf moisture measurement, as illustrated in Figure 10. Fabricated on a 0.8 mm F4B substrate, the antenna resonates between 2.40 and 3.0 GHz when unloaded and features a rectangular slot above the feed point for improved coupling and sensitivity. Sensitivity analysis, conducted for leaf permittivity values between 20 and 30, demonstrates the antenna’s superior performance compared with traditional designs. The experimental results show sensitivity increases of 0.57 to 1.67 times across various leaf samples, with modified and enhanced coupling designs exhibiting sensitivities of 0.06 GHz and 0.02 GHz, respectively. The system achieved high accuracy in moisture detection, with a low mean relative error of 0.038 between the predicted and measured moisture content. The effectiveness of the antenna as a moisture sensor, designed for real-time agricultural monitoring and precision farming, was validated through comprehensive analysis and modeling.

**Figure 11:**
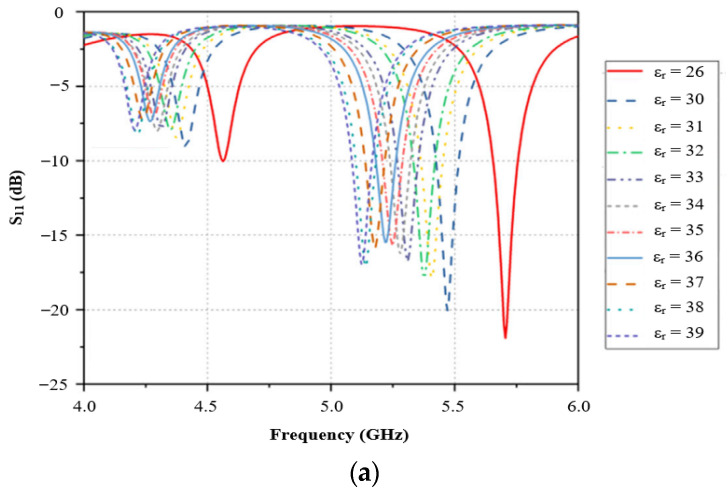

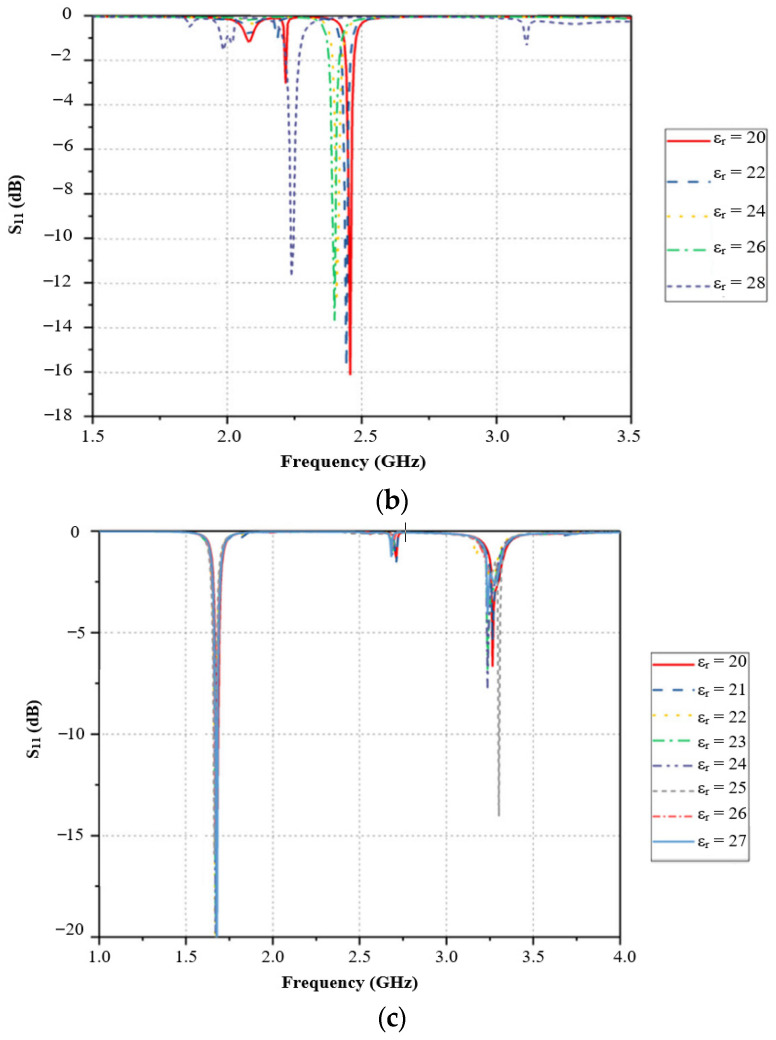
Return loss (dB) vs. frequency (GHz) for varying relative permittivity: (**a**) standard rectangular patch antenna, (**b**) slotted patch antenna, and (**c**) enhanced coupling modified slotted patch antenna [21].

The sensing mechanism is based on effective permittivity changes in the MW frequency range, enhanced by slotted microstrip patch antennas, which optimize the interaction with moisture in the leaves. This is further demonstrated by the return loss (dB) versus frequency (GHz) graphs for the varying relative permittivity values shown in Figure 11.

The enhanced performance of the new microstrip patch antenna design is primarily attributed to two key modifications: the incorporation of rectangular slots above the feed point, and the implementation of an advanced coupling technique. These changes significantly improve the sensitivity of the antenna for leaf moisture sensing. The slots alter the electrical characteristics of the antenna, allowing it to resonate at lower frequencies while maintaining a compact size. The advanced coupling technique enhances the interaction between the antenna and leaf sample, increasing the responsiveness of the sensor to changes in moisture content. To further improve the design, potential avenues could include optimizing the slot geometry for even greater sensitivity, exploring alternative substrate materials with better dielectric properties, and incorporating metamaterial structures to manipulate the electromagnetic field distribution. Additionally, enhancing IoT integration for real-time monitoring and developing more sophisticated data analysis algorithms could improve overall system performance and practical applicability in agricultural settings.

**Figure 12:**
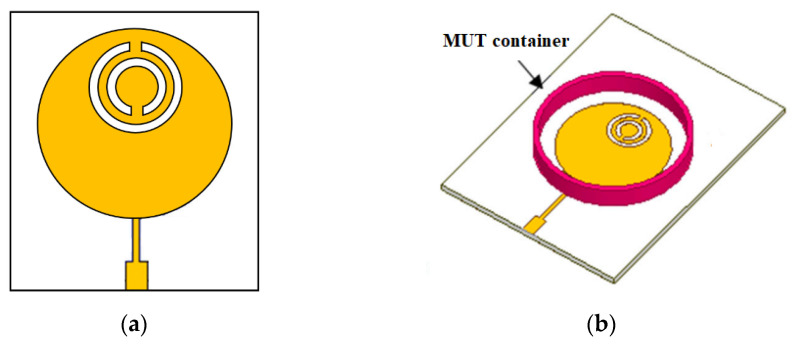
(**a**) Circular patch antenna, and (**b**) sensor structure with soil sample [23].

Oliveira et al. [23] developed a novel MW sensor for characterizing the relative permittivity of dielectric materials and determining water concentrations in soil samples. The sensor consisted of a circular microstrip patch antenna integrated with two slotted complementary split-ring resonators (CSRRs). The key features of the sensor include higher sensitivity compared to previous designs, effective resonance frequency shifts correlating with changes in water content, compact design, low cost, easy fabrication, and the requirement of a small material under test (MUT) sample, as shown in Figure 12a,b.

**Figure 13:**
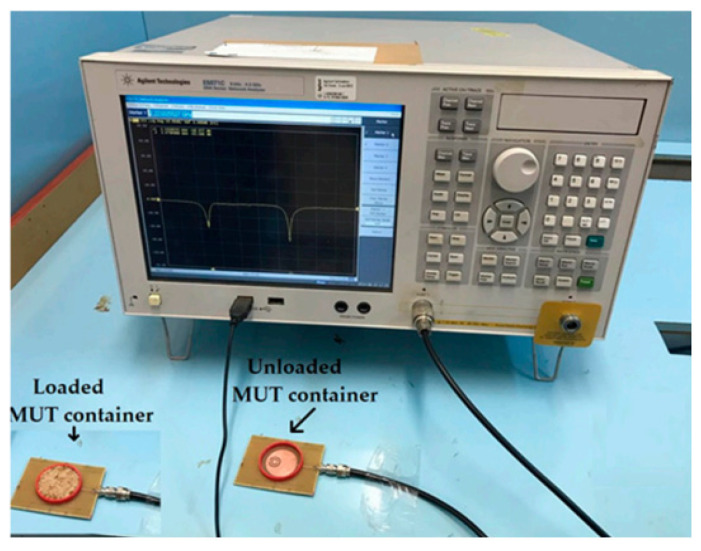
Fabricated sensor prototype and measurement setup [23].

The fabricated sensor prototype and its measurement setup are illustrated in Figure 13, demonstrating the practical implementation of the sensor for experimental validation.

**Figure 14:**
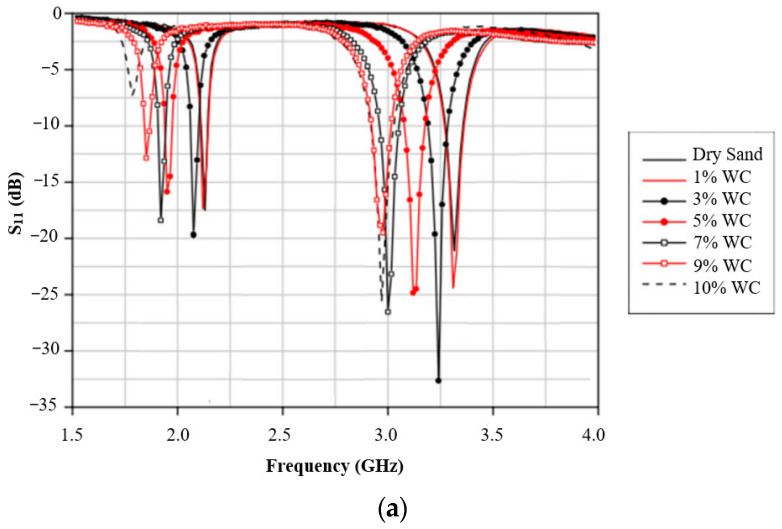

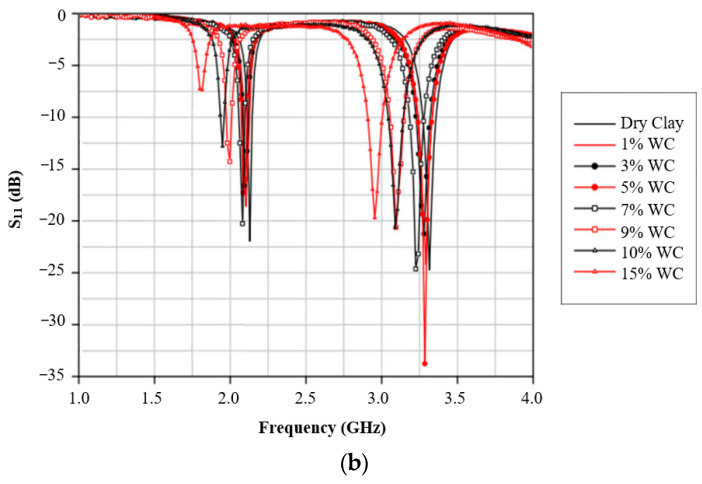
Return loss variation for different soil water content: (**a**) quartz sand and (**b**) red clay [23].

The sensor was tested on quartz sand with seven water concentrations (dry, 1%, 3%, 5%, 7%, 9%, and 10%) and on red clay with eight water concentrations (dry, 1%, 3%, 5%, 7%, 9%, 10%, and 15%). The return loss variations corresponding to different soil water contents for quartz sand and red clay are depicted in Figure 14a and Figure 14b, respectively, highlighting the sensor’s ability to detect moisture-induced dielectric changes.

The sensor operates by detecting shifts in resonant frequencies caused by the presence of soil samples, enabling applications across agriculture and environmental monitoring. This study contributes a modeling approach that determines the dielectric constant using dual resonances, applies the sensor for soil water content characterization, and achieves size reduction compared to earlier designs. The improved performance stems from the complementary split-ring resonator (CSRR) structure, which enhances sensitivity to variations in the surrounding medium’s permittivity. Dual-band operation provides multiple resonant frequencies, increasing measurement accuracy. An empirical model correlates each resonance frequency with the material’s permittivity, further refining characterization precision. The sensor’s compact, planar design also supports high sensitivity to small permittivity changes. Future enhancements could explore combining CSRRs with split-ring resonators (SRRs) to assess both permittivity and magnetic permeability, experimenting with substrate materials or thicknesses to extend sensitivity or frequency range, applying advanced data analysis techniques such as machine learning, miniaturizing the sensor without compromising performance, and integrating it with complementary measurement methods to broaden its applicability and accuracy.

Lira et al. [24] developed a compact MW sensor for measuring soil water content (SWC). The sensor was based on a rectangular microstrip antenna with a complementary split-ring resonator (CSRR) incorporated into its design. The sensor operates by detecting changes in the resonance frequencies owing to variations in the relative permittivity of the material under test (MUT). The authors described the design process, sensitivity analysis, and experimental validation of the sensor.

The sensor was designed and optimized to enhance the sensitivity. It features two resonance frequencies that shift in response to changes in the permittivity of the MUT. The authors conducted a thorough sensitivity analysis by comparing sensor performance with other designs from the literature. They also performed electric field analysis to optimize the CSRR dimensions for improved sensitivity.

The sensor was tested on Cambisol soil with ten water concentrations (0%, 1%, 3%, 5%, 7%, 9%, 11%, 13%, 15%, and 16%) and Latosol soil with nine water concentrations (0%, 1%, 3%, 5%, 7%, 9%, 11%, 13%, and 14%).

They derived empirical models to correlate the resonance frequencies with soil permittivity and SWC. The results showed good agreement between the simulated and measured data, with mean errors of 3.18% and 3.63% for the first and second resonance frequencies, respectively.

Ref. [24] presents a rectangular microstrip antenna integrated with a complementary split-ring resonator (CSRR) optimized through electric field analysis to enhance sensitivity relative to prior circular CSRR designs such as [23]. The dual-resonance operation, achieved by the CSRR incorporation, increases measurement accuracy by providing two distinct resonant frequencies. While previous studies have applied dual-resonance and CSRR structures for soil water content (SWC) characterization, the current work demonstrates that the square CSRR geometry enables improved sensitivity and frequency detection resolution compared to circular counterparts, as evidenced by comparative performance metrics. The optimization of CSRR dimensions tailored to the rectangular design further amplifies these benefits. These enhancements, alongside a detailed performance comparison with existing sensors, underscore the practical advantages of the proposed sensor in SWC measurement applications.

Several approaches can be considered to further improve sensor performance. Different CSRR geometries or arrangements can be explored to enhance the field concentration. Advanced materials with superior dielectric properties have been investigated as substrates. In addition, incorporating multiple CSRRs or exploring other metamaterial structures may lead to even greater sensitivity [62].

Recent developments in soil moisture sensing techniques using microstrip patch antennas have led to significant advancements in sensitivity, compactness, and versatility. These sensors, which are based on detecting changes in dielectric properties due to moisture variations, show promise for real-time agricultural monitoring and precision farming. The modified slotted microstrip patch antenna and circular microstrip patch antenna integrated with CSRRs both exhibited improved performance over traditional designs, with enhanced sensitivity and accuracy in measuring moisture content across various soil types and plant materials.

These innovations address key challenges in soil moisture sensing and offer the potential for more precise and efficient agricultural practices. However, further research is needed to optimize these designs, explore new materials and structures, and develop more sophisticated data analysis techniques. As this field continues to evolve, it has significant potential for advancing sustainable agriculture and environmental monitoring.

#### 2.5.2. Soil Moisture Resonator-Based Sensors

**Figure 15:**
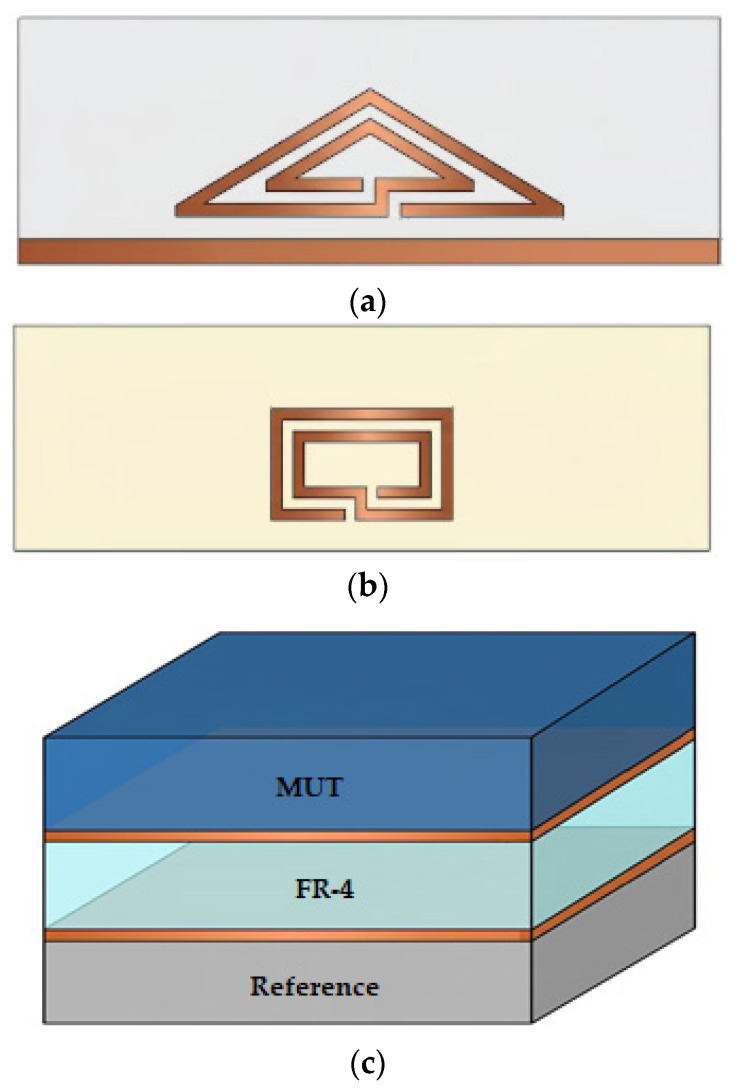
Proposed sensor: (**a**) top layer, (**b**) bottom layer, and (**c**) stackup schematic and materials.

Keshavarz et al. [25] introduced a highly sensitive differential soil moisture sensor (DSMS) to measure volumetric water content (VWC) in soil. The sensor uses a microstrip line loaded with two resonators: a triangular two-turn resonator (T2-SR) and complementary rectangular two-turn spiral resonator (CR2-SR). T2-SR is used to sense the dielectric properties of the soil, while CR2-SR serves as a reference to eliminate environmental effects. The sensor operates based on the principle that changes in soil moisture content affect its dielectric properties, which in turn influence the resonance frequency of T2-SR, as illustrated in Figure 15, showing the proposed sensor’s top layer, bottom layer, and stackup schematic with materials.

**Figure 16:**
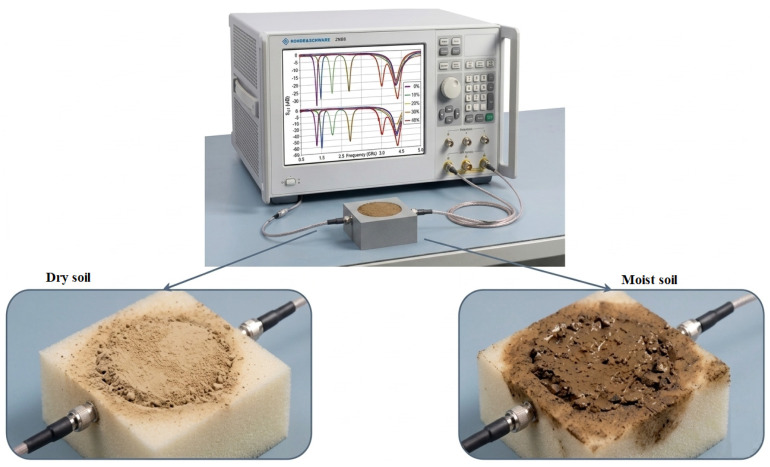
Measurement setup of sensor. Reproduced from [25].

The sensor exhibited a frequency shift of 110 MHz for a 1% change in soil moisture content at the highest level (30% VWC) for sandy-type soil. The operation of the sensor is explained through circuit model analysis and simulations. A prototype of the 3-cell DSMS was fabricated and tested to validate the differential sensing concept, with the measurement setup depicted in Figure 16.

The sensor demonstrates high sensitivity over a broad range of relative permittivities (1 to 16.5) and is capable of measuring soil moisture content from 0% to 30% VWC.

A unique aspect of this sensor is its differential measurement capability, which enhances its reliability by eliminating common-mode environmental factors. The use of both sides of the PCB to realize the two types of resonators results in a compact and low-cost design. The sensor’s performance is compared favorably with other recently reported permittivity measurement sensors, showing its high sensitivity and broad measurement range. The proposed DSMS proves the concept of differential MW sensing for precision agriculture applications and has the potential for use in other permittivity measurement scenarios.

**Figure 17:**
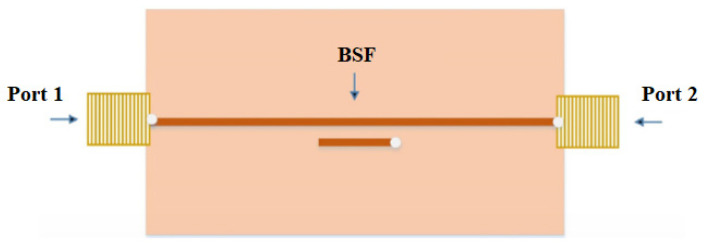
The configuration of the band stop filter sensor for the measurement of soil [56].

Karasaeng et al. [56] introduced an MW sensor to measure soil moisture using a microstrip band stop filter configuration, depicted in Figure 17. The sensor design incorporates parallel coupled lines on an FR4 substrate operating at 2.45 GHz. This study aimed to address the limitations of existing soil moisture measurement techniques by offering improved precision and cost-effectiveness.

**Figure 18:**
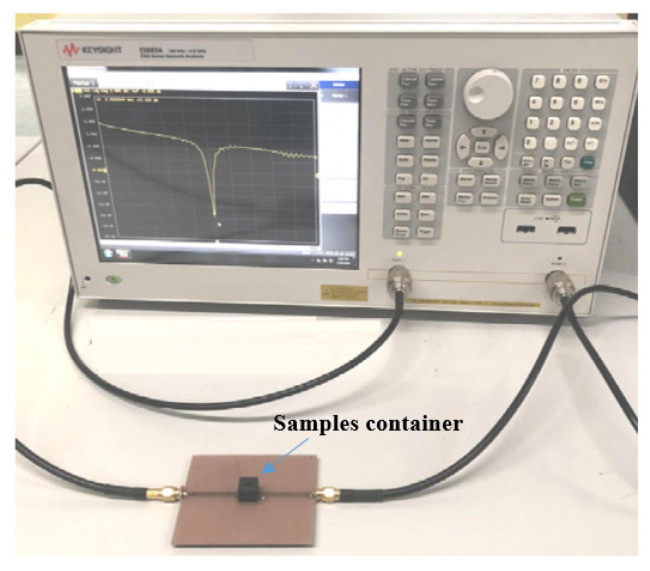
The experimental setup [56].

Their MW sensor was designed using microstrip parallel coupled lines with specific dimensions (width of 2.45 mm, spacing of 0.2 mm, and length of 17.06 mm) on an FR4 substrate. The sensor was tested using soil samples with moisture levels ranging from 0 to 100% in 20% increments. Measurements were conducted using a KEYSIGHT E5063A network analyzer operating in the frequency range of 100 MHz to 4.5 GHz, as illustrated in Figure 18.

**Figure 19:**
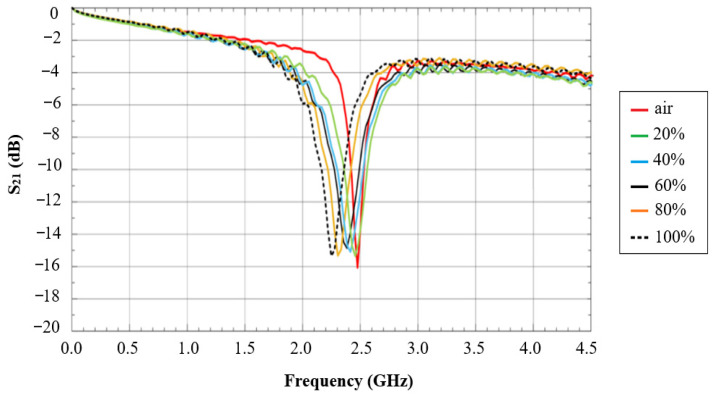
The measurement results of insertion loss ($S_{21}$) at different soil moisture levels [56].

The measurement results of the insertion loss at different soil moisture levels are presented in Figure 19. These results showed a clear correlation between the soil moisture levels and frequency shifts in the sensor response. As the soil moisture increased, the frequency of the insertion loss ($S_{21}$) decreased, with shifts ranging from 0 MHz for air (0% moisture) to 200 MHz for 100% soil moisture. The percentage differences in frequency shifts demonstrated a linear relationship with soil moisture levels ranging from 0% to 8.163%.

This study offers unique insights into the application of MW technology for soil moisture measurements. The use of a microstrip band stop filter configuration presents a novel approach to sensor design. The experimental results indicate that this design does offer improved accuracy and sensitivity compared with traditional methods. The linear relationship observed between the frequency shifts and soil moisture levels suggests that this sensor design could be particularly effective for precise soil moisture monitoring in agricultural and environmental applications. Additionally, the cost-effectiveness and ease of implementation of this sensor design make it a promising tool for widespread use in various fields that require accurate soil moisture data.

Yi et al. [57] presented a study on the development and application of an MW sensor system for measuring the soil moisture content and dielectric properties. The system operates in the frequency range of 2.2 GHz to 4.4 GHz and utilizes a microstrip ring resonator (MRR) sensor.

The MW sensor system consists of several components, including an MW controllable synthesizer, microstrip S-band MW directional coupler, power detectors, and data acquisition unit. The system measures the reflected and incident voltages to compute the return loss. The conducted experiments with soil samples of varying moisture content (0–26%) and observed frequency shifts in the resonance conditions as the moisture content changed.

The study also compared the results obtained from an MW sensor system with measurements from a commercial dielectric probe and vector network analyzer. Polynomial equations were developed to predict the moisture content, relative dielectric constant, and conductivity based on the observed frequency shifts. The system demonstrated good reliability and accuracy in determining the soil moisture content and dielectric properties.

The unique and interesting insights from this document include the system’s ability to distinguish between bound water and free water conditions in soil–water mixtures. Two distinct regions are identified in the return loss curves, corresponding to bound water “A” (18–20% moisture content) and free water “B” (22–26% moisture content) conditions. This distinction is significant for understanding soil–water interactions and could have important implications for agricultural applications. Additionally, the compact and portable nature of the developed MW sensor system, combined with the major advantage that it does not require a network analyzer, makes it a promising tool for in situ soil moisture measurements. This feature is particularly important for field use, offering practical advantages over traditional laboratory-based methods.

Recent advancements in MW sensing techniques using resonator-based sensor designs have led to significant progress in soil monitoring. The three studies discussed showcase innovative approaches for soil moisture measurement, each offering unique advantages.

The differential soil moisture sensor (DSMS) introduced by R. Keshavarz et al. [25] provides high sensitivity and reliability through its differential measurement capability. The microstrip band stop filter sensor developed by Karasaeng et al. [56] offers a cost-effective and precise solution with a clear linear relationship between the frequency shifts and soil moisture levels. Yi Lung et al.’s [57] MW sensor system, utilizing a microstrip ring resonator, demonstrated the ability to distinguish between bound and free water conditions in soil–water mixtures.

These developments represent important steps towards more accurate, efficient, and versatile soil moisture measurement techniques. The combination of high sensitivity, cost-effectiveness, and portability in these sensor designs holds great promise for widespread applications in agriculture, environmental monitoring, and related fields. As research in this area continues, we can expect further refinements and innovations that will enhance our ability to effectively monitor and manage soil moisture content.

Table 1 synthesizes recent advances [59,60,63,64,65,66] in planar microwave-based soil moisture sensors, focusing on configuration, sensitivity, accuracy (R^2^), soil type, and moisture range. This comparison highlights the diversity of designs and their performance across different soil and environmental conditions.

The multiturn complementary spiral resonator (MCSR) on microstrip exhibits the highest reported sensitivity of 2.05%, credited to its multiturn spiral geometry that increases the effective sensing area and enhances interaction with soil dielectric properties. This design likely improves detection resolution and moisture responsiveness. Although accuracy data are not specified, its applicability spans various soil types with a material under test (MUT) permittivity range of 1 to 23, reflecting broad adaptability [59]. However, the moisture content range remains unspecified, limiting comprehensive performance evaluation. Key advantages include high sensitivity and broad soil applicability, while limitations involve a lack of detailed calibration metrics, quality factor, and test condition data.

The defected ground structure (DGS)-based planar sensor, featuring a modified complementary split-ring resonator (CSRR), leverages enhanced electromagnetic field concentration to improve sensitivity and accuracy. It achieves a very high accuracy (R^2^ = 0.9998) and sensitivity of 1.83%, demonstrating excellent calibration and reliable moisture detection. Tested on fine sand, coarse sand, grit, and clay, with a moisture range of 7 to 15%, this sensor is well suited for moderate moisture detection in granular soils [60]. Key advantages are exceptional calibration precision and high accuracy, while limitations include a narrow moisture range, limited soil diversity, and missing quality factor and detailed environmental test conditions.

The self-similar fractal planar sensor (MPS) utilizes fractal geometry to increase surface complexity, facilitating multi-scale interaction with soil moisture and enabling measurement across a broad moisture range. While sensitivity is not reported, it attains high accuracy (R^2^ = 0.9771) and covers a full moisture spectrum from 0 to 100%, indicating versatility in diverse moisture conditions. This sensor is effective in soils with organic matter content, addressing complex soil matrices with heterogeneous dielectric properties [63]. Key advantages include broad moisture range coverage and good accuracy in complex soils; limitations involve missing sensitivity, quality factor, calibration method, and test condition details.

The planar cavity antenna sensor concentrates electromagnetic energy within a specific volume to enhance sensitivity in targeted soil layers. It shows moderate accuracy (R^2^ = 0.872) without sensitivity data. Tested on silty clay loam soils, it operates within a moisture content range of 1 to 45%, making it suitable for fine-textured soils with moderate moisture levels [64]. Key advantages are targeted application to fine-textured soils and moderate moisture detection; limitations include moderate accuracy, a lack of sensitivity and quality factor data, and incomplete calibration and measurement condition reporting.

The rotated self-similar fractal (R-SSF) planar sensor employs a rotated fractal pattern that may improve spatial resolution and electromagnetic field uniformity, leading to enhanced detection accuracy and range. It exhibits very high accuracy (R^2^ = 0.99939) and is applicable to sandy, loam, and clayey soils. Its moisture range extends from 0 to 75% of field capacity, demonstrating broad applicability in common agricultural soils [65]. Key advantages are very high accuracy and broad soil applicability; limitations include the absence of sensitivity, quality factor, calibration method, and test condition specifics.

Lastly, the reconfigurable complementary spiral resonator (CSR) integrated into a patch antenna offers tunability of sensor parameters to specific soil permittivity ranges (1–20), enhancing adaptability and potentially measurement precision. It presents a sensitivity of 1.7%, though accuracy and specific soil types or moisture ranges are not detailed, indicating a flexible but less characterized sensor design [66]. Key advantages include tunable sensor parameters for adaptability; limitations are incomplete accuracy, calibration, soil description, and test condition data.

Overall, the table underscores the varied balance between sensitivity, accuracy, operational range, and environmental adaptability among the sensor types. Spiral resonator sensors (MCSR and CSR) emphasize high sensitivity and permittivity adaptability but require more comprehensive calibration and environmental data. Fractal sensors (MPS and R-SSF) provide broad moisture coverage and high accuracy in complex soils yet lack full technical characterization. The DGS-based sensor excels in calibration precision within granular soils but is constrained by moisture range and soil diversity. The cavity antenna sensor suits fine-textured soils with moderate moisture but is less characterized technically.

In summary, these sensor configurations balance sensitivity, accuracy, and operational range differently. Spiral resonator designs (MCSR and CSR) prioritize high sensitivity and adaptability to permittivity variations, fractal sensors (MPS and R-SSF) provide broad moisture coverage and high accuracy in complex soils, the DGS-based sensor excels in calibration precision within granular soils, and the cavity antenna targets fine-textured soils with moderate moisture. Sensor selection should consider specific application needs regarding soil type, moisture range, and desired sensitivity and accuracy.

## 3. Challenges and Future Directions

Despite significant advancements in planar microwave (MW) soil moisture sensors, several critical challenges remain that impede their widespread adoption and effective field deployment. These challenges are further informed by the comparative performance characteristics summarized in Table 1, which highlights the diversity of sensor configurations, their sensitivity, accuracy, soil types tested, and moisture content ranges [59,60,63,64,65,66].

Planar microwave (MW) sensors exhibit spatial resolution that is primarily governed by the electromagnetic field distribution around the sensing structure, which is influenced by sensor geometry and operating frequency. Higher MW frequencies, closer to the upper end of the microwave band, correspond to shorter wavelengths, enabling finer spatial resolution and more localized sensing volumes. Conversely, lower MW frequencies provide deeper penetration into the soil but at the expense of coarser spatial resolution. The volume of soil effectively interrogated by these sensors is typically confined to the near-field region where the electric field is concentrated, often limited to a few millimeters to centimeters in depth and lateral extent for resonator-based sensors such as split-ring resonators (SRRs) and complementary split-ring resonators (CSRRs). Antenna-based sensors, such as microstrip patch antennas, can probe larger soil volumes due to their radiative near- and far-field patterns, with penetration depth dependent on frequency and antenna design. The depth of microwave penetration into soil is influenced by frequency, soil dielectric properties, and moisture content; lower frequencies penetrate deeper but reduce resolution, while higher frequencies enhance sensitivity and resolution but limit sensing to surface or near-surface layers. These factors create inherent trade-offs: increasing frequency improves sensitivity and spatial resolution but reduces penetration depth and sensing volume, while larger sensor structures increase soil volume coverage but may reduce sensitivity to localized changes and increase environmental noise susceptibility. Optimizing sensor design requires balancing spatial resolution, penetration depth, sensing volume, and sensitivity according to the specific application requirements, soil characteristics, and desired measurement depth in precision agriculture and environmental monitoring contexts.

(1) Sensitivity

Sensitivity varies notably among sensor designs, with the multiturn complementary spiral resonator (MCSR) on the FR4 microstrip substrate demonstrating the highest reported sensitivity of 2.05% [59]. This is attributed to its multiturn spiral geometry that enhances the sensing area and interaction with soil dielectric properties. The defected ground structure (DGS)-based planar sensor on Rogers RO3006 substrate achieves a sensitivity of 1.83% with near-perfect accuracy (R^2^ = 0.9998), indicating excellent moisture detection in granular soils such as fine sand, coarse sand, grit, and clay within a moisture range of 7 to 15% [60]. Fractal-based sensors, including the self-similar fractal planar sensor (MPS) on FR4 and the rotated self-similar fractal (R-SSF) planar sensor on Rogers RT/duroid 5880, prioritize broad moisture coverage (0–100% and 0–75% field capacity, respectively) with high accuracy (R^2^ = 0.9771 and 0.99939) but do not report explicit sensitivity values [63,65]. The reconfigurable complementary spiral resonator (CSR) in patch antenna on the Rogers RO4003C substrate presents moderate sensitivity (1.7%) with flexible permittivity range adaptability but lacks detailed accuracy and soil-specific data [66]. The variation in sensitivity and selectivity across these configurations highlights the challenge of optimizing sensor designs to achieve both high responsiveness and specificity across diverse soil types and moisture conditions.

(2) Accuracy

Accuracy, often reflected by the coefficient of determination (R^2^), varies significantly among sensors. The DGS-based planar sensor and the R-SSF planar sensor provide near-perfect accuracy (R^2^ > 0.999), demonstrating excellent calibration and reliable moisture detection in granular and common agricultural soils [60,65]. Conversely, the planar cavity antenna sensor shows moderate accuracy (R^2^ = 0.872) on silty clay loam soils within a moisture range of 1–45%, indicating limitations in precision for fine-textured soils [64]. The MCSR and CSR sensors lack specified accuracy data, limiting full performance evaluation [59,66]. These disparities underscore the need for advanced materials and fabrication techniques to enhance the quality factor (Q-factor), reduce energy losses, and improve sensor resolution and reliability.

(3) Multi-Parameter Sensing

Most existing planar MW sensors focus on single-parameter detection, primarily soil moisture, which restricts their versatility in complex agricultural environments. Although fractal sensors cover broad moisture ranges, the integration of multi-resonant structures and metamaterials for simultaneous detection of other soil parameters such as pH, salinity, and nutrient content remains limited. Future research should prioritize developing multi-parameter sensing platforms combined with advanced data interpretation algorithms, including machine learning, to enable comprehensive soil monitoring.

(4) Cost Efficiency

The choice of substrate material significantly influences both cost and performance. Sensors fabricated on low-cost FR4 substrates, such as the MCSR and MPS, offer affordability but may compromise on Q-factor and environmental robustness [59,63]. High-performance substrates like Rogers RO3006 and RT/duroid 5880, used in DGS-based and R-SSF sensors, provide superior electrical characteristics and accuracy but increase material costs [60,65]. The Rogers RO4003C substrate for the CSR sensor offers a balance but with less characterized performance [66]. This trade-off between cost and performance presents a barrier to large-scale agricultural deployment. Future efforts should explore cost-effective materials and scalable fabrication techniques that maintain sensor performance while reducing production expenses.

(5) Real-World Applicability

Environmental variability significantly impacts sensor reliability. Soil heterogeneity, including texture, organic matter, and particle distribution, affects dielectric properties and sensor readings. For instance, the DGS-based sensor was validated on granular soils with moisture content between 7 and 15%, while fractal sensors effectively cover soils with organic matter and broader moisture ranges [60,63]. This necessitates site-specific calibration curves, complicating large-scale deployment due to sensor-to-sensor variability and calibration drift. Temperature sensitivity further challenges accuracy, as soil temperature variations alter dielectric constants, potentially confounding moisture measurements. Differential sensor designs and compensation algorithms, such as artificial neural networks, offer promising approaches to mitigate these effects but require further development for robust field applications. Additionally, for long-term deployment in harsh soil environments, the use of protective coatings or encapsulation methods is essential to safeguard sensor components from moisture ingress, chemical corrosion, and mechanical damage. These protective measures help maintain sensor integrity and performance over extended burial periods, addressing durability concerns alongside calibration and environmental variability. Future research should focus on developing sensors with broader operating ranges, enhancing durability and longevity in harsh environments, and improving the calibration and maintenance protocols for long-term reliability.

(6) IoT Integration and Data Management

The integration of MW sensors into IoT networks and the management of large datasets present significant challenges. Future research in this area should include developing efficient data transmission and storage protocols, creating robust algorithms for real-time data analysis, and implementing secure and scalable IoT architectures for sensor networks [21,34,59]. These advancements will be crucial for leveraging the full potential of MW sensors in the context of IoT and big data applications.

To address these challenges, forthcoming research should emphasize the design and development of advanced sensor architectures explicitly engineered to fulfill the stringent requirements of passive sensing applications [5,6,7]. This initiative entails an in-depth investigation into novel material systems exhibiting superior electromagnetic characteristics, alongside systematic evaluation of diverse geometrical configurations [8] to maximize sensor sensitivity and spatial resolution. Concurrently, the progression of fabrication methodologies remains pivotal; state-of-the-art techniques such as additive manufacturing and three-dimensional (3D) printing offer substantial potential to streamline production workflows, reduce fabrication expenses, and facilitate the realization of complex antenna geometries previously unattainable through conventional processes.

In addition to hardware advancements, the enhancement of sophisticated signal-processing algorithms is critical [1,4]. These algorithms underpin the improved detection fidelity and classification accuracy of microwave (MW) sensing platforms by effectively mitigating noise, extracting salient features, and managing voluminous datasets. Machine learning paradigms, particularly those leveraging neural networks and deep learning frameworks [1,4], have demonstrated significant efficacy in deciphering intricate patterns embedded within high-dimensional electromagnetic signals, thereby enabling precise interpretation and robust decision-making.

## 4. Conclusions

Planar MW sensors represent a significant technological solution with demonstrated potential for non-invasive, real-time monitoring of crucial soil properties in agricultural applications. The pervasive trend toward using metamaterial-inspired structures has successfully addressed the critical need for miniaturization combined with deep soil sensing. Furthermore, the move toward differential and multi-band operation significantly enhances sensor robustness against environmental factors and reduces the need for constant recalibration. These advancements, when integrated into IoT architectures, enable true precision agriculture outcomes. By providing high-resolution, real-time data on soil moisture and nutrient availability, planar sensors allow farmers to implement optimal irrigation scheduling and targeted fertilizer application. This leads directly to substantial economic and environmental benefits, including documented water savings and increases in crop yields. Although challenges persist, notably in ensuring reliable performance in heterogeneous soils, compensating for temperature variations, and addressing the inherent sensor-to-sensor calibration drift, future research focusing on multi-parameter sensing, advanced signal processing, and integrated smart system design will ensure that planar permittivity MW sensors are poised to become powerful tools for optimizing agricultural practices, enhancing productivity, and promoting sustainable resource management in smart farming systems.

## Figures and Tables

**Table 1:** Summary of recent advances in planar microwave-based soil moisture sensors.

Configuration	Substrate	Operating Frequency (GHz)	Size (mm^2^)	Measurement Type	Sensitivity	Accuracy/Calibration (R^2^)	Soil Type	Moisture Content Range	Ref.
Multiturn complementary spiral resonator (MCSR) on microstrip	FR4	2.45	50 × 50	VNA-based	2.05%	Not specified	Various (MUT permittivity 1–23)	Not specified	[59]
DGS-based planar sensor (mod. CSRR)	Rogers RO3006	2.38	50 × 50	VNA-based	1.83%	0.9998	Fine sand Coarse sand Grit Clay	7 to 15%	[60]
Self-similar fractal planar sensor (MPS)	FR4	2.4	30 × 30	VNA-based	Not specified	0.9771	Soil with organic matter content	0–100%	[63]
Cavity antenna (planar)	Not specified	1.5–3	96 × 46	VNA-based	Not specified	0.872	Silty Clay Loam	1–45%	[64]
Rotated self-similar fractal (R-SSF) planar sensor	Rogers RT/duroid 5880	2.4	60 × 50	VNA-based	Not specified	0.99939	Sandy, loam, clayey	0–75% field capacity	[65]
Reconfigurable complementary spiral resonator (CSR) in patch antenna	Rogers RO4003C	0.95–0.97	50 × 50	VNA-based	1.7%	Not specified	Permittivity 1–20	Not specified	[66]

## Data Availability

No new data were created or analyzed in this study.

## References

[1] Karthikeyan L., Pan M., Wanders N., Kumar D.N., Wood E.F. (2017). Four decades of microwave satellite soil moisture observations: Part 1. A review of retrieval algorithms. Adv. Water Resour..

[2] Akash M., Mohan Kumar P., Bhaskar P., Deepthi P.R., Sukhdev A. (2024). Review of estimation of soil moisture using active microwave remote sensing technique. Remote Sens. Appl. Soc. Environ..

[3] Njoku E.G., Entekhabi D. (1996). Passive microwave remote sensing of soil moisture. J. Hydrol..

[4] Wigneron J.-P., Jackson T.J., O’Neill P., De Lannoy G., de Rosnay P., Walker J.P., Ferrazzoli P., Mironov V., Bircher S., Grant J.P. (2017). Modelling the passive microwave signature from land surfaces: A review of recent results and application to the L-band SMOS & SMAP soil moisture retrieval algorithms. Remote Sens. Environ..

[5] Philippe J., De Paolis M.V., Arenas-Buendia C., Henry D., Coustou A., Rumeau A., Aubert H., Pons P. (2018). Passive and chipless packaged transducer for wireless pressure measurement. Sens. Actuators A Phys..

[6] El Matbouly H., Boubekeur N., Domingue F. (2015). Passive Microwave Substrate Integrated Cavity Resonator for Humidity Sensing. IEEE Trans. Microw. Theory Tech..

[7] Guha S., Jamal F.I., Wenger C. (2017). A Review on Passive and Integrated Near-Field Microwave Biosensors. Biosensors.

[8] Khair N.S., Talip Yusof N.A., Wahab Y.A., Bari B.S., Ayob N.I., Zolkapli M. (2023). Substrate-integrated waveguide (SIW) microwave sensor theory and model in characterising dielectric material: A review. Sens. Int..

[9] Wang C., Ali L., Meng F.-Y., Adhikari K.K., Zhou Z.L., Wei Y.C., Zou D.Q., Yu H. (2021). High-Accuracy Complex Permittivity Characterization of Solid Materials Using Parallel Interdigital Capacitor- Based Planar Microwave Sensor. IEEE Sens. J..

[10] Hanif A., Alam T., Tariqul Islam M., Albadran S., Alsaif H., Alshammari A.S., Alzamil A. (2025). Compact Complementary Highly Sensitive Microwave Planer Sensor for Reliable Material Dielectric Characterization. IEEE Sens. J..

[11] Ali L., Wang G., Kumar Adhikari K., Khan I., Cheng Y.-F., Wang C. (2024). A CSRR-Based Microwave Sensor for Characterizing Multiple Magneto-Dielectric Materials. IEEE Sens. J..

[12] Ali L., Wang C., Meng F.-Y., Adhikari K.K., Gao Z.-Q. (2022). Interdigitated Planar Microwave Sensor for Characterizing Single/Multilayers Magnetodielectric Material. IEEE Microw. Wirel. Compon. Lett..

[13] Alahnomi R.A., Zakaria Z., Yussof Z.M., Althuwayb A.A., Alhegazi A., Alsariera H., Rahman N.A. (2021). Review of Recent Microwave Planar Resonator-Based Sensors: Techniques of Complex Permittivity Extraction, Applications, Open Challenges and Future Research Directions. Sensors.

[14] Pourafzal A., Roi-Taravella T., Cheffena M., Yayilgan S.Y. (2023). A Low-Cost and Accurate Microwave Sensor System for Permittivity Characterization.

[15] Oliveira J.G.D., Junior J.G.D., Pinto E.N.M.G., Neto V.P.S., D’Assunção A.G. (2020). A New Planar Microwave Sensor for Building Materials Complex Permittivity Characterization. Sensors.

[16] Kaur S., Singh S., Sinha M.M. (2024). Prototype of Circular Split Ring Resonator-Based Sensor for Estimating Soil Moisture as a Function of Soil Particle Distribution. IEEE Sens. J..

[17] Ye N., Walker J.P., Yeo I.-Y., Jackson T.J., Kerr Y., Kim E., Mcgrath A., Popstefanija I., Goodberlet M., Hills J. (2021). Toward P-Band Passive Microwave Sensing of Soil Moisture. IEEE Geosci. Remote Sens. Lett..

[18] Salski B., Czekala P., Krupka J., Kopyt P. (2022). A Microwave Sensor of Moisture Content and Salinity of Soil. IEEE Sens. J..

[19] Silva L.A.P., de Assis Brito Filho F., de Andrade H.D. (2023). Soil Moisture Monitoring System Based on Metamaterial-Inspired Microwave Sensor for Precision Agriculture Applications. IEEE Sens. J..

[20] Abdolrazzaghi M., Nayyeri V., Martin F. (2022). Techniques to Improve the Performance of Planar Microwave Sensors: A Review and Recent Developments. Sensors.

[21] Khan M.T., Lin X.Q., Zhe C., Saboor A. (2024). Design, analysis and validation of a microstrip patch antenna with enhanced coupling for leaf moisture sensing: An IoT approach. Front. Phys..

[22] Felix W.C., de Oliveira A.H.S., de Amorim Junior R., Nobre D.B., de Oliveira Souto L., da Silva V.E., Felix R.A. (2020). Soil Moisture Sensor with a Microstrip Band Stop Filter. Proceedings of the 2020 IEEE 6th World Forum on Internet of Things (WF-IoT).

[23] Oliveira J.G.D., Pinto E.N.M.G., Silva Neto V.P., D’Assunção A.G. (2020). CSRR-Based Microwave Sensor for Dielectric Materials Characterization Applied to Soil Water Content Determination. Sensors.

[24] de Andrade Lira R.V., Freire C.R., Da Silva I.B.T., da Silva Neto V.P., de Oliveira J.G.D., de Andrade H.D., de Siqueira Campos A.L.P. (2024). A compact CSRR-based microwave sensor for soil water content. Sens. Actuators A Phys..

[25] Keshavarz R., Lipman J., Schreurs D.M.M.-P., Shariati N. (2021). Highly Sensitive Differential Microwave Sensor for Soil Moisture Measurement. IEEE Sens. J..

[26] Ong N.T.J., Yee S.K., Ashyap A.Y.I. (2020). Design of Microwave Sensor Based on Rectangular Double Split Ring Resonator for Water Quality Monitoring. Proceedings of the 2020 IEEE Student Conference on Research and Development (SCOReD).

[27] Akhir S.A.M., Ibrahim S.Z., Rosli N., Zain A.S.M., Khalid N. (2019). Antenna for humidity sensor using split ring resonator. Indones. J. Electr. Eng. Comput. Sci..

[28] Saeidi T., Alhawari A.R.H., Almawgani A.H.M., Alsuwian T., Imran M.A., Abbasi Q. (2022). High Gain Compact UWB Antenna for Ground Penetrating Radar Detection and Soil Inspection. Sensors.

[29] Herrmann P.S.D.P., Sydoruk V., Marques Porto F.N. (2020). Microwave Transmittance Technique Using Microstrip Patch Antennas, as a Non-Invasive Tool to Determine Soil Moisture in Rhizoboxes. Sensors.

[30] Fauziah M., Pramudita A.A., Nugroho B.S. (2023). Extraction Formula for Microstrip Antenna as Soil Water Content Sensor. Proceedings of the 2023 IEEE International Symposium On Antennas And Propagation (ISAP).

[31] Javadizadeh S., Badieirostami M., Shahabadi M. (2023). Ultrasensitive miniaturized planar microwave sensor for characterization of water–alcohol mixtures. Sci. Rep..

[32] Frau I., Wylie S., Byrne P., Onnis P., Cullen J., Mason A., Korostynska O. (2021). Microwave Sensors for In Situ Monitoring of Trace Metals in Polluted Water. Sensors.

[33] Palandoken M., Gocen C. (2025). Microwave sensor designs for liquid material dielectric characterization: Technological advances and applications. Sens. Actuators A Phys..

[34] Chang Z., Zhang F., Xiong J., Ma J., Jin B., Zhang D. (2022). Sensor-free Soil Moisture Sensing Using LoRa Signals. Proc. ACM Interact. Mob. Wearable Ubiquitous Technol..

[35] Javanbakht N., Xiao G., Amaya R.E. (2021). A Comprehensive Review of Portable Microwave Sensors for Grains and Mineral Materials Moisture Content Monitoring. IEEE Access.

[36] Priyaa A.S.P., Mohammed A., Ambili C., Anusree N.S., Thekekara A.V., Mohan R.R., Mridula S. (2025). Microwave Sensor Antenna for Soil Moisture Measurement. Proceedings of the 2015 Fifth International Conference on Advances in Computing and Communications (ICACC).

[37] Yin H., Cao Y., Marelli B., Zeng X., Mason A.J., Cao C. (2021). Soil Sensors and Plant Wearables for Smart and Precision Agriculture. Adv. Mater..

[38] Kozak R., Khorsand K., Zarifi T., Golovin K., Zarifi M.H. (2021). Patch antenna sensor for wireless ice and frost detection. Sci. Rep..

[39] Li X., Xue S., Xie L., Wan G. (2024). A miniaturized passive wireless patch antenna sensor for structural crack sensing. Struct. Health Monit..

[40] Balanis C.A. (2016). Antenna Theory: Analysis and Design.

[41] Yeo J., Lee J.-I. (2019). Slot-Loaded Microstrip Patch Sensor Antenna for High-Sensitivity Permittivity Characterization. Electronics.

[42] Liu Q., Deng H., Meng P., Sun H. (2021). High Sensitivity Sensor Loaded With Octagonal Spiral Resonators for Retrieval of Solid Material Permittivity. IEEE Sens. J..

[43] Al-Behadili A.A., Mocanu I.A., Codreanu N., Pantazica M. (2020). Modified Split Ring Resonators Sensor for Accurate Complex Permittivity Measurements of Solid Dielectrics. Sensors.

[44] Ma J., Tang J., Wang K., Guo L., Gong Y., Wang S. (2021). Complex Permittivity Characterization of Liquid Samples Based on a Split Ring Resonator (SRR). Sensors.

[45] Ye W., Wang D.-W., Wang J., Wang G., Zhao W.-S. (2022). An Improved Split-Ring Resonator-Based Sensor for Microfluidic Applications. Sensors.

[46] Javed A., Arif A., Zubair M., Mehmood M.Q., Riaz K. (2020). A Low-Cost Multiple Complementary Split-Ring Resonator Based Microwave Sensor for Contactless Dielectric Characterization of Liquids.

[47] Gan H.-Y., Zhao W.-S., Liu Q., Wang D.-W., Dong L., Wang G., Yin W.-Y. (2020). Differential Microwave Microfluidic Sensor Based on Microstrip Complementary Split-Ring Resonator (MCSRR) Structure. IEEE Sens. J..

[48] Ds C., Nagini K.B.S.S., Barik R.K., Koziel S. (2024). Highly Sensitive Microwave Sensors Based on Open Complementary Square Split-Ring Resonator for Sensing Liquid Materials. Sensors.

[49] Su L., Mata-Contreras J., Vélez P., Fernández-Prieto A., Martín F. (2018). Analytical Method to Estimate the Complex Permittivity of Oil Samples. Sensors.

[50] Al-Gburi A.J.A., Zakaria Z., Abd Rahman N., Alam S., Said M.A.M. (2023). A Compact and Low-Profile Curve-Feed Complementary Split-Ring Resonator Microwave Sensor for Solid Material Detection. Micromachines.

[51] Haq T., Ruan C., Zhang X., Ullah S., Fahad A.K., He W. (2020). Extremely Sensitive Microwave Sensor for Evaluation of Dielectric Characteristics of Low-Permittivity Materials. Sensors.

[52] Haq T., Ruan C., Zhang X., Kosar A., Ullah S. (2019). Low cost and compact wideband microwave notch filter based on miniaturized complementary metaresonator. Appl. Phys. A.

[53] Sharafadinzadeh N., Abdolrazzaghi M., Daneshmand M. (2020). Investigation on planar microwave sensors with enhanced sensitivity from microfluidic integration. Sens. Actuators A Phys..

[54] Saadat-Safa M., Nayyeri V., Khanjarian M., Soleimani M., Ramahi O.M. (2019). A CSRR-Based Sensor for Full Characterization of Magneto-Dielectric Materials. IEEE Trans. Microw. Theory Tech..

[55] Gulsu M.S., Bagci F., Can S., Yilmaz A.E., Akaoglu B. (2021). Minkowski-like fractal resonator-based dielectric sensor for estimating the complex permittivity of binary mixtures of ethanol, methanol and water. Sens. Actuators A Phys..

[56] Karasaeng W., Nualkham J., Summatta C., Sonasang S. (2023). Measurement of Soil Moisture Using Microwave Sensors Based on BSF Coupled Lines. Eng. Proc..

[57] Then Y.L., You K.Y., Dimon M.N. (2014). Soil moisture dielectric measurement using microwave sensor system. Proceedings of the 2014 International Symposium on Antennas and Propagation Conference Proceedings.

[58] Kumar P., Chaturvedi A. (2020). Design and Development of Single & Dual Resonant Frequency Antennas for Moisture Content Measurement. Wirel. Pers. Commun..

[59] Raza A., Keshavarz R., Shariati N. (2024). Precision Agriculture: Ultra-Compact Sensor and Reconfigurable Antenna for Joint Sensing and Communication (Version 2). arXiv.

[60] Pereira R.N., Júnior J.G.D., Santana Praxedes M.E.T., Cabral K.C., da Silva Neto V.P., D’Assunção A.G. (2024). A planar DGS sensor for moisture analysis in civil construction aggregates. Sens. Actuators A Phys..

[61] Wu X., He H., Liao T., Xu H., Lu G., Wu Z. (2024). Agricultural Drought Monitoring Using an Enhanced Soil Water Deficit Index Derived from Remote Sensing and Model Data Merging. Remote Sens..

[62] Alsaif H., Islam M.S., Hoque A., Islam M.R., Islam M.T., Soliman M.S. (2024). Dual circular complementary split ring resonator based metamaterial sensor with high sensitivity and quality factor for textile material detection. APL Mater..

[63] Kaur S., Singh S., Sinha M.M., Rajendran D., Kanoun O. (2025). A Low-Profile Self-Similar Geometry-Based Microwave Planar Sensor for Assessing the Impact of Organic Matter Content on Soil Field Capacity. IEEE Sens. J..

[64] Iaccheri E., Berardinelli A., Tartagni M., Ragni L. (2024). Affordable Microwave Soil Moisture Detector. IEEE Sens. J..

[65] Kaur S., Singh S., Sinha M.M. (2025). Design and Fabrication of a Low-Profile Planar Sensor for the Estimation of Plant-Available Water in Different Textured Soils. IEEE Trans. Geosci. Remote Sens..

[66] Raza A., Keshavarz R., Dutkiewicz E., Shariati N. (2023). Compact Multiservice Antenna for Sensing and Communication Using Reconfigurable Complementary Spiral Resonator. IEEE Trans. Instrum. Meas..

